# Functional Role of Odorant-Binding Proteins in Response to Sex Pheromone Component *Z*8-14:Ac in *Grapholita molesta* (Busck)

**DOI:** 10.3390/insects15120918

**Published:** 2024-11-25

**Authors:** Yuqing Luo, Xiulin Chen, Shiyan Xu, Boliao Li, Kun Luo, Guangwei Li

**Affiliations:** 1 Shaanxi Province Key Laboratory of Jujube, College of Life Science, Yan’an University, Yan’an 716000, China; 13981374642@163.com (Y.L.); chenxiulin@yau.edu.cn (X.C.); liboliao@yau.edu.cn (B.L.); luok@yau.edu.cn (K.L.); 2 Shaanxi Province Fruit Industry Research and Development Center, Xi’an 710000, China; xsy-2121@163.com

**Keywords:** *Grapholita funebrana*, *Grapholita molesta*, sex pheromone, inhibition, fluorescence competitive binding assay

## Abstract

The plum fruit moth (PFM), *Grapholita funebrana*, and the oriental fruit moth (OFM), *G. molesta*, are closely related fruit moth species that severely damage fruit trees in Rosaceae. Both species share common primary sex pheromone components *Z*8-12:Ac and *E*8-12:Ac. The secondary sex pheromone components of PFMs consist of *Z*8-12:OH, *Z*8-14:Ac, and *Z*10-14:Ac, while those of OFMs include *Z*8-12:OH and 12:OH. Previous researchers have proved that the inclusion of *Z*8-14:Ac and *Z*10-14:Ac did not augment PFM catches but inhibited OFM catches in orchards in Europe, thereby maintaining the species-specificity of the PFM sex attractant. However, which of these components, *Z*8-14:Ac or *Z*10-14:Ac, plays the major role in inhibiting OFM attraction remains unclear. In the current study, electroantennogram (EAG) assays indicated that both OFM and PFM males exhibited a moderate EAG response to *Z*8-14:Ac and *Z*10-14:Ac. Rubber septa loaded with varying ratios of *Z*8-14:Ac (1% to 30%) or *Z*10-14:Ac (5% to 110%) combined with a constant dose of *Z*8-12:Ac and *E*8-12:Ac produced diverse trapping effects. Sex attractants containing *Z*8-14:Ac did not significantly affect the trapping of PFM males but drastically reduced the capture of OFM males, with the reduction reaching up to 96.54%. Attractants containing more than 10% of *Z*10-14:Ac simultaneously reduced the number of OFM and PFM males captured. *Z*8-14:Ac was indispensable for maintaining the specificity of sex pheromones. Fluorescence competitive binding assays of recombinant GmolPBP2 showed the lowest *K_i_* value (0.66 ± 0.02 μM) among the PBPs/GOBPs from OFMs, suggesting that it is the most likely target for *Z*8-14:Ac. Molecular dynamic simulation and site-directed mutagenesis assays confirmed that the Phe12 residue, which forms a π–alkyl interaction with *Z*8-14:Ac, was crucial for GmolPBP2 binding to *Z*8-14:Ac. In conclusion, *Z*8-14:Ac is vital to the specificity of PFM sex pheromones inhibiting OFM attractants when added to *Z*8-12:Ac and *E*8-12:Ac. This could be potentially used to develop species-specific sex attractants for the PFM.

## Simple Summary

This study investigates the inhibitory effect of sex pheromone components of the plum fruit moth (PFM), *Grapholita funebrana*, loaded with different doses of (*Z*)-8-tetradecenyl acetate (*Z*8-14:Ac) or (*Z*)-10-tetradecenyl acetate (*Z*10-14:Ac) on the trapping of the closely related species, the male oriental fruit moth (OFM), *G*. *molesta*. Field tests showed that adding 5–30% of *Z*8-14:Ac to a mixture of *Z*8-12:Ac and *E*8-12:Ac did not significantly affect the trapping of PFM males but reduced OFM male trapping by more than 86%. In contrast, adding over 10% of *Z*10-14:Ac to a mixture of *Z*8-12:Ac and *E*8-12:Ac inhibited the trapping of both OFM and PFM males. GmolPBP2 from OFMs exhibited the strongest binding affinity for *Z*8-14:Ac compared to the other GmolPBP/GmolGOBP and was considered the most likely OBP for recognizing and transporting *Z*8-14:Ac. Mutating the Phe12 residue of GmolPBP2 to Ala12 resulted in a loss of affinity for *Z*8-14:Ac, suggesting that Phe12 was the key amino acid and that π–alkyl was the primary weak interaction maintaining the binding affinity for GmolPBP2. These findings enhance our understanding of the molecular mechanisms through which insects recognize the sex pheromones of closely related species and thereby contribute to the development of species-specific sex attractants for PFMs using secondary sex pheromones.

## 1. Introduction

The oriental fruit moth (OFM), *Grapholita molesta,* and the plum fruit moth (PFM), *G. funebrana* (Lepidoptera: Tortricidae), are important fruit-boring pests worldwide, causing serious economic losses to stone and pome fruits [1,2]. Previous studies assumed that OFM larvae primarily feed on peaches, pears, and apples [3,4,5], whereas PFM larvae mainly consume plums and apricots [6,7]. However, over the past two decades, with the expansion of apple, peach, and pear cultivation in Northwest China, the PFM population in orchards has rapidly increased, becoming the dominant species in regions such as Xinjiang, Shaanxi, and Gansu Provinces of China [8,9,10]. The sympatric distribution of PFMs and OFMs, along with their similar external morphologies, makes it difficult to distinguish them [11]. Moreover, the cross-attraction between the commercial sex attractants for PFMs and OFMs affects the accuracy of estimating moth populations in peak occurrences [12,13], leading to misjudgment in monitoring their occurrence periods and population sizes.

The attractiveness of a sex attractant primarily relies on the integrity of the female pheromones and the ratio of each pheromone component [14,15]. Each moth species employs a unique blend of pheromones to attract the conspecific males while inhibiting males of the closely related heterospecific species. Secondary components play a crucial role in enhancing the species specificity of sex pheromones, thereby maintaining reproductive isolation [16,17]. The sex pheromones of PFMs consist of (*Z*)-8-dodecenyl acetate (*Z*8-12:Ac), (*E*)-8-dodecenyl acetate (*E*8-12:Ac), (*Z*)-8-dodecen-1-ol (*Z*8-12:OH), (*Z*)-8-tetradecenyl acetate (*Z*8-14:Ac), and (*Z*)-10-tetradecenyl acetate (*Z*10-14:Ac) at a 100:1:2:30:5 ratio (m/m) [18]. The sex pheromones of OFMs include *Z*8-12:Ac, *E*8-12:Ac, *Z*8-12:OH, and 1-dodecanol (12:OH) in ratios of 100:4.2-7.2:1.1-19.1:5.4-12.0 [19,20,21,22]. Currently, commercial sex attractants for PFMs contain a 100:4 blend of *Z*8-12:Ac and *E*8-12:Ac, while those for the OFM are loaded with a 100:6:1-2 blend of *Z*8-12:Ac, *E*8-12:Ac, and *Z*8-12:OH [18,23,24]. The cross-attraction and the lack of species-specificity can be attributed to the shared major components and the absence of secondary components in their sex attractants [25]. An excellent study by Guerin et al. (1986) discovered that the inclusion of *Z*8-14:Ac and *Z*10-14:Ac did not augment PFM catches but inhibited OFM catches in plum orchards in Switzerland and in peach orchards bordered by plums in Hungary [18]. However, following studies did not determine how *Z*8-14:Ac and *Z*10-14:Ac from PFM females inhibit OFM male attractants.

It is widely recognized that odorant binding proteins (OBPs) are essential in detecting and transporting hydrophobic volatile semiochemicals from antennae pores to olfactory receptors [26,27]. The OBP family proteins were originally categorized into pheromone-binding proteins (PBPs), general odorant-binding proteins (GOBPs), and antennal-binding proteins (ABPs) based on their differences in binding odorant molecules [28]. *PBPs* show biased expression in male antennae and are distributed in long sensilla trichodea, where they function in recognizing and transporting sex pheromones and their analogs. Apart from Crambidae species, which usually possess four or more PBPs, the PBP subfamily typically consists of three members, namely, PBP1, PBP2, and PBP3 [29,30,31,32,33]. GOBPs, mainly localized at s. basiconca and short s. trichodea are equivalently expressed in both sexes in adult antennae, which are primarily involved in detecting and transporting host plant-derived volatiles and sex pheromones [33,34,35]. With the rapid expansion in genome and transcriptome sequencing techniques, an increasing number of studies have shown that GOBPs and PBPs are often clustered on the same chromosome in close proximity, suggesting that they might have derived from a single ancestral gene and then diverged through gene duplication events and various environmental selection pressures to function in detecting and recognizing sex pheromones, host plant volatiles, and even insecticides [36,37,38,39,40,41]. In our prior research, we identified three PBPs (GmolPBP1, GmolPBP2, and GmolPBP3) and two GOBPs (GmolGOBP1 and GmolGOBP2) in the antennae of the OFM using RNA-seq and RT-PCR [42]. Fluorescence competitive binding assays revealed that recombinant GmolPBPs and GmolGOBPs have different affinities for binding to sex pheromones [43,44,45]. Although *Z*8-14:Ac and *Z*10-14:Ac are not components of OFM sex pheromones, the roles of PBPs and GOBPs in OFMs when *Z*8-14:Ac and *Z*10-14:Ac are added to OFM lures remain unclear.

In the present study, we employed an electroantennogram (EAG) system to evaluate OFM and PFM males responding to individual sex pheromone components of OFMs and PFMs, including *Z*8-12:Ac, *E*8-12:Ac, *Z*8-12:OH, *Z*8-14:Ac, and *Z*10-14:Ac, at different doses. Then, we conducted field trapping trials to assess how OFMs respond when different doses of *Z*8-14:Ac or *Z*10-14:Ac are added to a mixture of *Z*8-12:Ac and *E*8-12:Ac. A high dose of *Z*10-14:Ac was found to inhibit both OFM and PFM male attraction when added to a mixture of *Z*8-12:Ac and *E*8-12:Ac (500:20) in pear orchards. In addition, GmolPBP2 was found to be the primary OBP responsible for recognizing and transporting *Z*8-14:Ac using fluorescence competition binding assays. Finally, the key amino acid residues and weak interactions involved in GmolPBP2 binding to *Z*8-14:Ac were identified via homology modeling, molecular dynamics simulations, and site-directed mutagenesis. This study advances our understanding of how moths recognize interspecific sex pheromones at the molecular level and provides data for developing species-specific PFM sex attractants using secondary sex pheromones.

## 2. Materials and Methods

### 2.1. Colony Maintenance

All individuals utilized in the experiment originated from a laboratory colony at Yan’an University in Yan’an, Shaanxi Province, China. The larvae were raised on an artificial diet [46] and kept in an artificial climate chamber maintained at 25 ± 1 °C and a relative humidity of 70 ± 5%, with a photoperiod (light/dark) of 15 h:9 h. Pupae of different sexes were separated, and newly emerged virgin males were placed in disposable plastic cups (4.0 cm bottom diameter, 6.5 cm top diameter, and 6.6 cm height) covered with a plastic film containing air vents. Adults were fed a 5% (*v*/*v*) honey solution until they were utilized for testing. Mature PFM larvae were gathered from the fruits of the flowering plum *Amygdalus triloba* surrounding the apple orchards in Yan’an in June 2023. After the larvae left their fruits, they were moved to moist sandy soil for pupation and adult emergence. The feeding conditions and methods used for PFM adults were the same as those used for OFMs.

### 2.2. Chemicals

The synthetic pheromones *Z*8-12:Ac, *E*8-12:Ac, and *Z*8-12:OH were sourced from Pherobio Technology Co., Ltd. (Yangling, China), while *Z*8-14:Ac and *Z*10-14:Ac were synthesized by Shenyang Beixinjingyi Trade Co., Ltd. (Shengyang, China). Chemical purity ranging from 95% to 98% and isomeric purity exceeding 98% were determined through gas chromatography (GC) (Agilent, Santa Clara, CA, USA) analysis. Additionally, the fluorescence probe N-Phenyl-1-naphthylamine (1-NPN) with a purity of 98% was obtained from Sigma-Aldrich (Milwaukee, WI, USA).

### 2.3. Electroantennogram Analysis

Electroantennogram (EAG) equipment (Syntech, Hilversum, The Netherlands) was used to record the antennal responses of the OFM and PFM adults to *Z*8-12:Ac, *E*8-12:Ac, *Z*8-12:OH, *Z*8-14:Ac, and *Z*10-14:Ac. These sex pheromone compounds were diluted with liquid paraffin to prepare a stimulus solution of 100 μg/μL, which was subsequently diluted to 10 and 1 μg/μL. Antenna from 3-day-old OFM and 2- to 4-day-old PFM virgin males were excised at its base, with the distal part of the terminal segment being removed. Then, the treated antenna was attached to electrode holders with SpectraR360 conducting gel. A stimulus controller (model CS-55, Syntegon Technology, Waiblingen, Germany) continuously supplied a humidified air stream at a rate of 500 mL/min. Then, 20 μL of each stimulus solution was adsorbed onto a twice-folded filter paper strip (0.5 cm × 4 cm) placed on the wide section of a l mL pipette tip, which served as an odorant cartridge. Vapor stimuli were delivered by an air compressor into the constant air stream described above, flowing in a copper delivery tube (i.d. 8 mm) with the outlet positioned approximately 1 cm from the antenna. The EAG amplitude tests of the OFM and PFM males’ responses to sex pheromones started with solvent control stimuli (20 μL of liquid paraffin), followed by stimulation with 20, 200, and 2000 μg doses (20 μL of 1.0, 10.0, and 100.0 μg/μL of liquid paraffin solutions, respectively) of stimulus in sequence. The solvent control was performed at the end of the run. To control for variations among antennae, each sample was tested on 10 antennae of different males. The EAG signals and data were analyzed using EAG 2000 software (Syntech, Hilversum, The Netherlands). The corrected EAG amplitude value = absolute EAG amplitude value − (EAG_ck1_ + EAG_ck2_)/2, where EAG_ck1_ and EAG_ck2_ represent the absolute EAG amplitude values of the solvent control before and after measuring each stimulus, respectively. The relative EAG response of the OFM and PFM males to the same doses of sex pheromones were subjected to an analysis of independent sample *t*-test (*p* = 0.05) using SPSS version 23.0 (IBM).

### 2.4. Determination of the Inhibitory Effects of Z8-14:Ac and Z10-14:Ac on OFM Males

To determine the inhibitory effects on OFM male adults and the impact on the trapping effects of PFM male adults, varying amounts of *Z*8-14:Ac (0, 5, 10, 25, 50, 75, 100, 125, 150 μg) or *Z*10-14:Ac (0, 25, 50, 100, 150, 250, 350, 450, and 550 μg) were added to rubber septa loaded with a constant dosage of the main pheromone (500 μg of *Z*8-12:Ac and 20 μg of *E*8-12:Ac) of the PFM. Trapping experiments of *Z*8-14:Ac were carried out in peach and pear orchards in Yan’an and Xinjiang (China), respectively. Trapping tests of *Z*10-14:Ac were performed in pear orchards in Xinjiang (China). Each orchard had nine plots, with three plots in one field. Each plot consisted of a linear arrangement of traps loaded with a constant dosage of the main pheromones and different percentages of the secondary pheromone *Z*8-14:Ac or *Z*10-14:Ac of the PFM. Plots in the same field were placed 30 m apart, and fields were ≥1 km apart. The traps were hung 1.4 m from the ground on a suspension bracket between two rows of peach or pear trees from 10 June to 25 July 2021 in Xinjiang and from 15 June to 30 July 2021 in Yan’an. The positions of the traps within the plot were randomly arranged. Every 5 days, counts were taken, and the traps were rotated to the adjacent position to avoid any potential position effects. The septa loaded with sex pheromone components were replaced with new ones after every 15 days of suspension. The data were analyzed using a one-way ANOVA, and the differences in the average trapping of OFM and PFM males using sex attractants containing different amounts of *Z*8-14:Ac or *Z*10-14:Ac were further analyzed using Tukey’s HSD test, both of which were performed using SPSS 23.0.

### 2.5. Tissue Expression of GmolPBPs and GmolGOBPs in OFM Adults

Tissue samples of antennae, head (without antennae), thorax, abdomen, legs, and wings were collected from 3-day-old virgin female and male adults. We utilized 200, 30, 20, 6, 100, and 100 females or males for antenna, head (without antennae), thorax, abdomen, leg, and wing samples for RNA isolation, respectively. Each sample had three independent biological replicates. The total RNA from all samples was isolated using an AG RNAex Pro reagent (AG, Changsha, China), and then the first-strand cDNA of each sample was synthesized from 2.0 μg of total RNA with EasyScript One-Step gDNA Removal and cDNA Synthesis SuperMix (TransGen Biotech, Beijing, China). Seven pairs of specific primers, including for five target genes *GmolPBP1*, *GmolPBP2*, *GmolPBP3*, *GmolGOBP1*, *GmolGOBP2*, and two reference genes *β-actin* (GenBank No. KF022227.1) and Elongation factor 1-α (*EF1-α*) (GenBank No. KT363835.1) were designed and synthesized (Table S1). The cycle threshold (Ct) and amplification efficiency (E) values of the target and reference genes in different tissues of the OFM adults were detected on a StepOnePlus Real-Time PCR Instrument (ABI, Carlsbad, CA, USA) using PerfectStart Green qPCR SuperMix (TransGen Biotech, Beijing, China). Their expression levels in each sample were calculated using Equation 1 from the study [47]. Finally, the normalized expression levels of *GmolPBP1–3* and *GmolGOBP1–2* in different tissues were calculated with the geometric mean of the expression of the two reference genes using Equation (2) from the study [47]. Due to the inhomogeneity of data variance, we transformed the data using the formula log_10_(*x* + $\sqrt{x^{2}+1}$), where *x* represents the normalized expression level of target genes, following the method described by Kelmansky et al. [48]. Differences in expression levels in different tissues were analyzed using ANOVA followed by Tukey’s HSD test in SPSS (SPSS v23.0, IBM). Significant differences in expression levels between the sexes in the same tissue of adults were tested using an independent *t*-test.

### 2.6. Preparation of rGmolPBPs/rGmolGOBPs Proteins and Fluorescence Competitive Binding Assays

Previous researchers have successfully expressed rGmolPBP1, rGmolPBP2, rGmolPBP3, rGmolGOBP1, and rGmolGOBP2 proteins using prokaryotic expression systems [43,44,45]. In this article, the DNA sequences of these five GmolPBPs and GmolGOBPs, with the signal peptides removed and restriction sites on both ends, were inserted into the expression vector pET28a(+) linearized with the same restriction enzyme. These vectors were then transformed into *Escherichia coli* BL21(DE3) and induced by isopropyl-β-d-thiogalactoside (IPTG). Since all five recombinant proteins were expressed in inclusion bodies, they were denatured using 8 M urea and then renatured using a cysteine/cystine REDOX system [49]. The reduced recombinant proteins were purified using an Ni-NTA His Bind Resin column (7Sea Pharmatech Co., Ltd., Shanghai, China). Non-target proteins were washed off with washing buffer (20 mM Tris-HCl (pH 7.4), 20 mM imidazole, 1 mM PMSF, and 250 mM NaCl), and then the target proteins were eluted with elution buffer (20 mM Tris-HCl (pH 7.4), 1 mM PMSF, and 250 mM NaCl) with an increasing concentration of imidazole (50, 100, and 200 mM). Five milliliters of eluents with high target protein content and low levels of contaminants were collected and placed in dialysis tubing (Coolaber, Beijing, China) with a molecular weight cut-off of 3.5 kDa to remove NaCl and imidazole. The purified soluble proteins were stored at −80 °C before use.

The binding affinities of three rGmolPBPs and two rGmolGOBPs for *Z*8-14:Ac were determined using an F-2700 fluorescence spectrophotometer (Hitachi, Tokyo, Japan). First, we measured the dissociation constants (*K_d_*) of the rGmolPBPs (or rGmolGOBPs) binding to 1−NPN. Each protein was diluted to 2 μM with 20 mM Tris-HCl buffer (pH 7.4); then, 2 mL of the protein solution was transferred to a quartz cuvette and titrated with 1 mM 1−NPN to a final concentration of 1–18 μM. As the doses of 1−NPN were increased, the increasing amplitude of the fluorescence intensity decreased, and finally, the binding of the protein to 1−NPN reached saturation. The *K_d_* values were calculated using the nonlinear regression method and the Scatchard equation. Second, we measured the inhibitory constant (*K_i_*) of *Z*8-14:Ac’s competitive binding with each rGmolPBP (or rGmolGOBP) from the protein/1−NPN complex. The initial fluorescence intensity values were measured after a 2 min reaction in 2 mL solution of 20 mM Tris-HCl (pH 7.4) containing 2 µM rGmolPBP (or rGmolGOBP) protein and 2 µM 1−NPN. Then, the solution was titrated with aliquots of 1 mM *Z*8-14:Ac to obtain final concentrations in the range of 1–14 µM, and the fluorescence intensity value of each reaction was recorded. The *K_i_* values were calculated using the following equation:

*K_i_* = [*IC*_50_]/(1 + [1 − *NPN*]/*K_d_*)

where *IC*_50_ is the concentration of *Z*8-14:Ac when half of the initial fluorescence intensity value of the rGmolPBP (or GmolGOBP)/1−NPN complex is replaced, [1 − *NPN*] represents the concentration of free 1−NPN, and *K_d_* is the dissociation constant of the rGmolPBP (or GmolGOBP)/1−NPN complex.

### 2.7. Homology Modeling and Molecular Docking

The fluorescence competitive binding assays revealed that GmolPBP2 was the most likely target for recognizing and transporting *Z*8-14:Ac; therefore, a three-dimensional (3D) model of GmolPBP2 was constructed and molecular dynamics simulations were used to predict the important amino acid residues and interaction forces between GmolPBP2 and *Z*8-14:Ac using the predicted GmolPBP2 interaction with the major sex pheromone *Z*8-12:Ac model for comparison. The crystal structure templates of GmolPBP2 were screened in the RCSB PDB (https://www.rcsb.org/, accessed on 1 April 2023). On the basis of the identity and coverage between the GmolPBP2 sequence and template sequences, the X-ray diffraction crystal structure of *Amyelois transitella* AtraPBP1 complexed with the sex pheromone component *Z*11, *Z*13-16:Ald (4INW) was utilized as a template to construct a homology model of GmolPBP2. EasyModeller 4.0 was employed to generate nine coarse homologous models of GmolPBP2. The discrete optimized protein energy (DOPE) method was used to evaluate the energy of each model. The model with the lowest energy was selected as the optimized model for GmolPBP2. The amino acid residues that form the binding pocket of GmolPBP2 were predicted using the PROTEINS PLUS online program (https://proteins.plus/, accessed on 15 April 2023). UCLA-DOE LAB—SAVES v6.0 online server (https://saves.mbi.ucla.edu/, accessed on 10 April 2023) was used to evaluate the quality of the optimized model of GmolPBP2. The 3D structure files of *Z*8-14:Ac (Compound CID: 5363518) and *Z*8-12:Ac (Compound CID: 5363377) were downloaded from the PubChem database (https://pubchem.ncbi.nlm.nih.gov/, accessed on 15 April 2023). Molecular docking of the GmolPBP2 interaction with *Z*8-14:Ac and *Z*8-12:Ac was performed using AutoDockTools-1.5.6 software with the default parameters. The top-ranked binding mode (with the lowest binding energy score) was chosen and visualized using PyMOL (v. 2.5.2). The 2D structure of predicted amino acid residues, as well as the interaction forces between GmolPBP2 and *Z*8-14:Ac and *Z*8-12:Ac, were visualized using the Discovery Studio Visualizer software 2016.

### 2.8. Molecular Dynamic Simulation and Calculation of Binding Free Energy of Each Amino Acid

The molecular dynamic (MD) simulations of GmolPBP2–*Z*8-14:Ac and GmolPBP2–*Z*8-12:Ac complexes were performed for 200 nanoseconds (ns), employing GROMACS (Version 2022.2) and AMBER99SB protein forcefield. The simulations started with the solvation of complexes to neutralize the system, followed by energy with the steepest descent algorithm with a step length of 0.01 nm. The systems were equilibrated under NVT (canonical ensemble) and NPT (isobaric–isothermal ensemble) for 2 ns. After that, a 200 ns simulation with a time step of 20 fs was performed. Analyses of the root-mean-square deviation (RMSD), root-mean-square fluctuation (RMSF), and radius of gyration (Rg) were executed using GROMACS (v. 2022.2) built-in tools [50] and visualized using the ggplot2 package (v. 3.4.3) in R (v. 4.3.2) [51]. Finally, the binding free energy of each amino acid of GmolPBP2 to *Z*8-14:Ac and *Z*8-12:Ac was calculated using the gmx_Molecular Mechanic and Poisson–Boltzmann Surface Area (gmx-MMPBSA) tool [52].

### 2.9. Site-Directed Mutagenesis and the Binding Affinities of GmolPBP2 Mutants to Z8-14:Ac

Five amino acids—Phe12, Leu68, Ile94, Arg109, and Ile113—were identified as having lower binding free energies for GmolPBP2 interaction with *Z*8-14:Ac. To assess their role in binding, these residues were individually mutated to alanine using site-directed mutagenesis. Primers for mutagenesis were designed following the protocols recommended by the Fast Mutagenesis System (TransGen Biotech, Beijing, China) (Table S1). These mutations were introduced into the pET-28a(+)–GmolPBP2 plasmid using a Fast Mutagenesis System kit (Vazyme, Nanjing, China) and site-directed mutagenesis primers generating the mutants F12A, L68A, I94A, R109A, and I113A, respectively. Each plasmid was verified by DNA sequencing to confirm the presence of the desired mutation. Expression and purification of the mutated proteins followed the same protocols used for the wild-type GmolPBP2. The binding affinities of the five mutants for *Z*8-14:Ac were determined via fluorescence competitive binding assays. An increase in *K_i_* values for the mutants relative to the wild-type GmolPBP2 would indicate that the corresponding amino acid residues may be crucial for binding to *Z*8-14:Ac.

## 3. Results

### 3.1. EAG Response of OFM and PFM Males to Different Doses of Sex Pheromones

**Figure 1:**
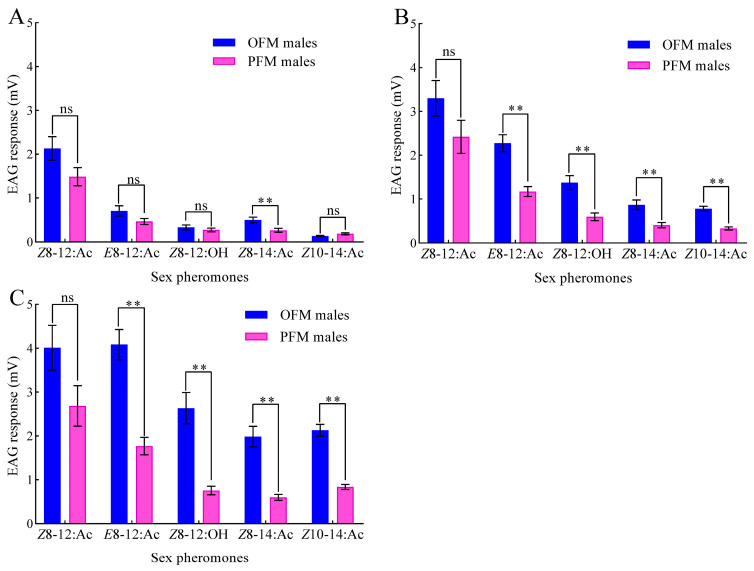
EAG dose–response of OFM and PFM males to different sex pheromone components: (**A**) 20 ng; (**B**) 200 ng; (**C**) 2000 ng. Data are presented as mean ± *SEM* (*n* = 10). Double asterisks and “ns” indicate extremely significant differences (*p* < 0.01) and no significant differences (*p* > 0.05), respectively, in the EAG response values between OFM and PFM males to the same sex pheromone (independent sample *t*-test).

There is a dose-dependent relationship between the EAG response values of OFM and PFM males to five sex pheromones: *Z*8-12:Ac, *E*8-12:Ac, *Z*8-12:OH, *Z*8-14:Ac, and *Z*10-14:Ac (Figure 1). Both OFM and PFM males exhibit a higher sensitivity to the primary components *Z*8-12:Ac and *E*8-12:Ac at the same dose compared to their response to the secondary components *Z*8-14:Ac and *Z*10-14:Ac (Figure 1). Surprisingly, the EAG values of the OFM males to different doses of *Z*8-14:Ac stimulation were significantly higher than those of the PFM males (20 μg: *t* = 2.959, *df* = 18, *p* = 0.008; 200 μg: *t* = 3.784, *df* = 18, *p* = 0.001; 2000 μg: *t* = 5.708, *df* = 18, *p* < 0.001). Except for the absence of significant difference in EAG response to 20 μg of *Z*10-14:Ac stimulation between OFM and PFM males (*t* = −1.865, *df* = 18, *p* = 0.079), the EAG values of OFM males to 200 and 2000 μg of *Z*10-14:Ac was significantly greater than that of PFM males (200 μg: *t* = 6.892, *df* = 18, *p* < 0.001; 200 μg: *t* = 8.809, *df* = 18, *p* < 0.001).

### 3.2. Effects of Z8-14:Ac and Z10-14:Ac on Captured OFM and PFM Males

The field tests using delta traps baited with the two primary PFM pheromones and different doses of the secondary sex pheromone *Z*8-14:Ac showed that adding *Z*8-14:Ac to a mixture of *Z*8-12:Ac and *E*8-12:Ac inhibited OFM males but not PFM males in both the peach and pear orchard trials (Table 1 and Table 2). Adding 1–30% of *Z*8-14:Ac to the PFM sex attractants neither significantly increased nor inhibited the number of PFM males in the peach (*F* = 1.893, *df* = 8,26, *p* = 0.124) and pear orchards (*F* = 1.072, *df* = 8,26, *p* = 0.424). Compared to the mixture of *Z*8-12:Ac and *E*8-12:Ac (100:4), adding 1–2% of *Z*8-14:Ac significantly reduced the capture of OFM males by 58.81–63.45% in the peach orchards (1% *Z*8-14:Ac: *t* = 9.358, *df* = 4, *p* = 0.001; 2% *Z*8-14:Ac: *t* = 10.650, *df* = 4, *p* < 0.001) (Table 1) and by 63.82–65.04% in the pear orchards (1% *Z*8-14:Ac: *t* = 7.806, *df* = 4, *p* = 0.001; 2% *Z*8-14:Ac: *t* = 8.873, *df* = 4, *p* = 0.001) (Table 2). In both the peach and pear orchard trials, the number of OFM males caught by adding 5–30% of *Z*8-14:Ac drastically reduced (by more than 86%) and were not significantly different among the sex attractants that contained varying amounts of *Z*8-14:Ac (peach orchard trails/pear orchard trails: *F* = 2.546, *df* = 5,17, *p* = 0.094; *F* = 1.639, *df* = 5,17, *p* = 0.224). In contrast to *Z*8-14:Ac, adding more than 10% of *Z*10-14:Ac to the mixture of *Z*8-12:Ac and *E*8-12:Ac (100:4) inhibited the capture of both OFM and PFM males, with reductions of up to 87.77% and 71.07%, respectively (Table 3). Therefore, *Z*8-14:Ac combined with a mixture of *Z*8-12:Ac and *E*8-12:Ac, but not *Z*10-14:Ac, can serve as a specific component for PFM sex attractants to inhibit OFM male adults.

### 3.3. Tissue Expression of GmolPBPs and GmolGOBPs

**Figure 2:**
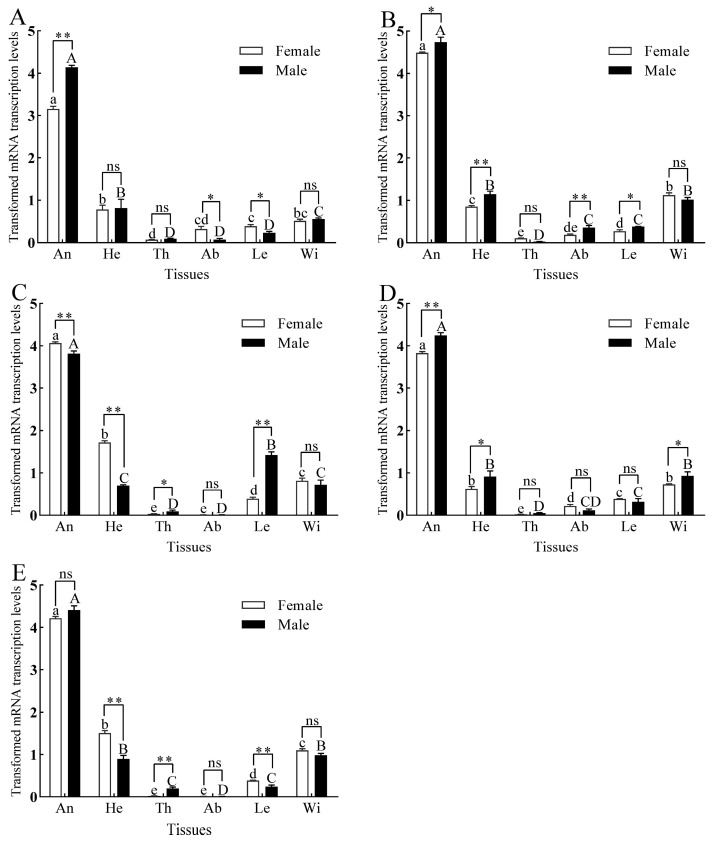
Tissue expression patterns of three *GmolPBPs* and two *GmolGOBPs*. (**A**) *GmolPBP1*. (**B**) *GmolPBP2*. (**C**) *GmolPBP3*. (**D**) *GmolGOBP1*. (**E**) *GmolGOBP2*. Different lowercase and capital letters above each bar indicate significant differences in relative transcript levels of male and female adults across various tissues, as determined by one-way ANOVA followed by Tukey’s HSD test (*p* < 0.05). Double asterisks, single asterisks, and “ns” indicated extremely significant differences (*p* < 0.01), significant differences (*p* < 0.05), and no significant difference (*p* > 0.05), respectively, in expression levels between males and females within the same tissue (independent sample *t*-test).

All three *GmolPBPs* and two *GmolGOBPs* were predominantly expressed in the antennae of both male and female adults, with slight expression in the heads, wings, and legs and minimal expression in the thoraxes and abdomens (Figure 2). The expression levels of *GmolPBP1*, *GmolPBP2*, and *GmolGOBP1* were significantly higher in the male antennae than in the female antennae (Figure 2A,B,D), whereas the expression of *GmolPBP3* was notably higher in the female antennae compared to the male antennae (Figure 2C). No significant difference in expression was observed for *GmolGOBP2* between male and female antennae (Figure 2E). Beyond the antennae, sex-specific differences in expression were also observed in other tissues. For instance, in the head, *GmolPBP3* and *GmolGOBP2* expression levels were significantly higher in the females (Figure 2C,E), whereas *GmolPBP2* and *GmolGOBP1* levels were significantly higher in males (Figure 2B,D). In the legs, the expression of *GmolPBP1* and *GmolGOBP2* were higher in females (Figure 2A,E), while the expression of *GmolPBP2* and *GmolPBP3* were higher in males (Figure 2B,C). Notably, the expression of *GmolGOBP1* in male wings was significantly elevated compared to female wings (Figure 2D), while no significant sex differences were observed for the other four *GmolPBPs*/*GmolGOBPs* in wings (Figure 2A–C,E).

### 3.4. Binding Affinities of rGmolPBPs and rGmolGOBPs for Z8-14:Ac

**Figure 3:**
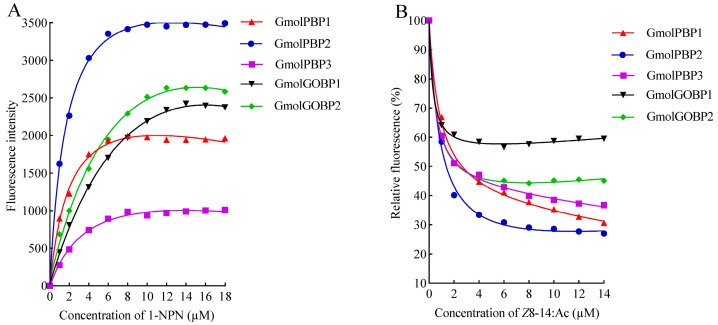
Curves of rGmolPBPs and rGmolGOBPs binding to 1−NPN probe (**A**) and sex pheromone *Z*8-14:Ac (**B**). In (**A**), each rGmolPBP and rGmolGOBP protein was diluted to a concentration of 2 µM with 20 mM Tris-HCl buffer (pH 7.4), and then aliquots of 1−NPN were added to reach a final concentration of 1 to 18 µM. The calculated *K_d_* values of rGmolPBP1, rGmolPBP2, rGmolPBP3, rGfunGOBP1, and rGmolGOBP2 were 1.29 ± 0.02, 1.09 ± 0.02, 11.24 ± 0.08, 5.76 ± 0.01, and 4.32 ± 0.02 µM, respectively. In (**B**), the fluorescence intensity after *Z*8-14:Ac displaced the 1−NPN probe bound to rGmolPBPs and rGmolGOBPs is displayed as a percentage of the initial fluorescence intensity. The calculated *K_i_* values of the rGmolPBPs and rGmolGOBPs with *Z*8-14:Ac are listed in Table 4.

We successfully expressed and purified rGmolPBP1, rGmolPBP2, rGmolPBP3, rGmolGOBP1, and rGmolGOBP2 proteins using an *E*. *coli* BL21(DE3) expression system (Figure S1). Fluorescence saturation was observed in these five proteins when titrated with increasing concentrations of the fluorescent probe 1−NPN, indicating the suitability of 1−NPN for measuring their binding affinities for odorant ligands (Figure 3A). The calculated *K_d_* values of 1−NPN interacting with rGmolPBP1, rGmolPBP2, rGmolPBP3, rGmolGOBP1, and rGmolGOBP2 were 1.29 ± 0.02, 1.09 ± 0.02, 11.24 ± 0.08, 5.76 ± 0.01, and 4.32 ± 0.02 μM, respectively (Table 4). The *K_i_* value of rGmolGOBP1 for *Z*8-14:Ac could not be calculated because 50% inhibition was not reached, and an *IC_50_* value could not be extrapolated. The other four proteins showed strong binding affinities for *Z*8-14:Ac, with *K_i_* values of 1.66 ± 0.04, 0.66 ± 0.02, 2.56 ± 0.27, and 2.86 ± 0.18 μM, respectively (Figure 3B). The lowest *K_i_* value for rGmolPBP2 binding to *Z*8-14:Ac suggests that it is the most likely target for *Z*8-14:Ac, indicating a better fit in the GmolPBP2 binding pocket, resulting in stronger interactions with the protein.

### 3.5. Homology Modeling and Molecular Dynamics Simulation of GmolPBP2

**Figure 4:**
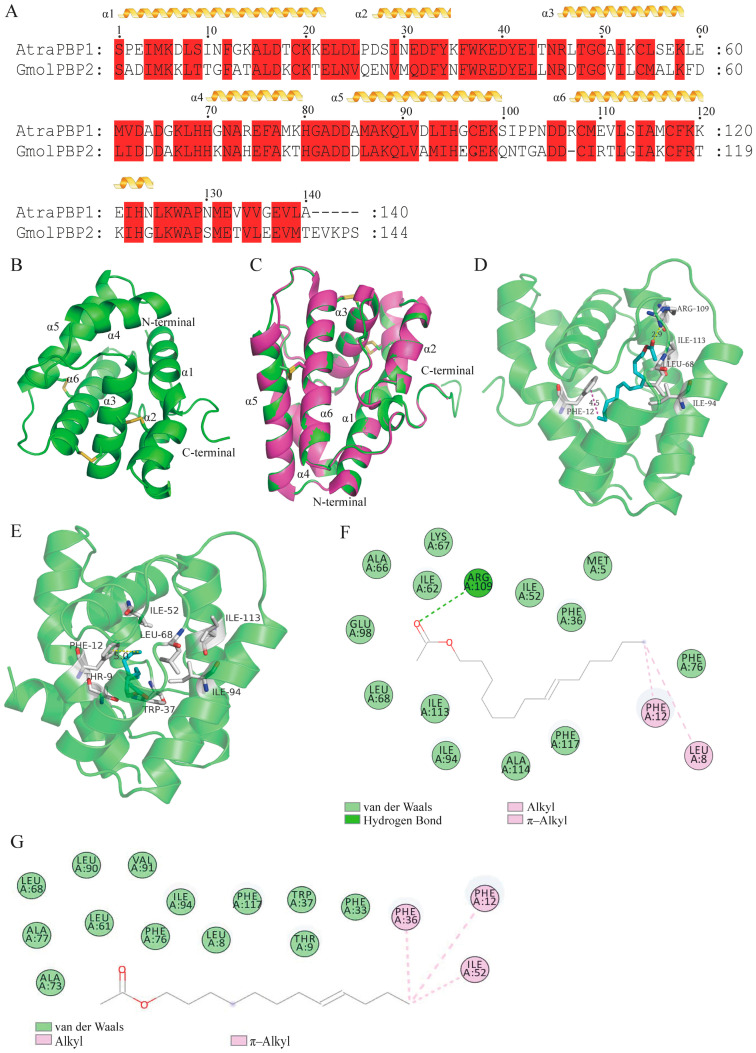
Construction of a 3D model of GmolPBP2 and analysis of the amino acid residues and interaction forces in GmolPBP2–*Z*8-14:Ac and GmolPBP2–*Z*8-12:Ac. (**A**) Sequence alignment of GmolPBP2 and AtraPBP1. α-helices are displayed as squiggles, while residues that are strictly identical are highlighted with a red background. (**B**) Predicted 3D model of GmolPBP2. (**C**) Structure alignment of AtraPBP1 (4INW) and GmolPBP2. (**D**,**E**) represent the predicted amino acid residues and interaction forces of GmolPBP2 binding to *Z*8-14:Ac and *Z*8-12:Ac, respectively (3D structure). (**F**,**G**) represent the predicted amino acid residues and interaction forces of GmolPBP2 binding to *Z*8-14:Ac and *Z*8-12:Ac, respectively (2D structure).

The 3D structure of GmolPBP2 was constructed using the X-ray diffraction crystal structure of AtraPBP1 from the *A. transitella* complex (4INW) as a template (Figure 4B,C). A *BLASTP* search revealed a 53.96% amino acid similarity between GmolPBP2 and AtraPBP1, with a sequence coverage of 97% (Figure 4A). Ramachandran plots indicated that 94.0% of the residues in GmolPBP2 were situated in the favorable region, while 6.0% were in the allowed region, indicating a high quality of this model (Figure S2). The predicted 3D structure of GmolPBP2 featured six typical α-helices located between residues Ser1-Glu22 (α1), Glu27-Tyr34 (α2), Arg46-Lys58 (α3), His70-Thr79 (α4), Asp84-Gln100 (α5), and Asp106-G123 (α6). Three pairs of disulfide bridges connected Cys19 in α1 and Cys54 in α3, Cys50 in α3 and Cys107 in α6, and Cys97 in α5 and Cys116 in α6 (Figure 4B). It was found that 33 amino acids, including Met5, Leu8, Thr9, Phe12, Phe33, Phe36, Trp37, Ile52, Leu53, Met55, Ala56, Leu61, Ile62, Ala66, Lys67, Leu68, Ala73, His74, Phe76, Ala77, Leu86, Ala87, Leu90, Ala91, Ile94, Glu98, Arg109, Thr110, Ile113, Ala114, Phe117, Arg118, and Val133, were involved in the formation of the hydrophobic binding pockets of GmolPBP. The proportion of hydrophobic amino acids was 78.79% (Table S2).

**Figure 5:**
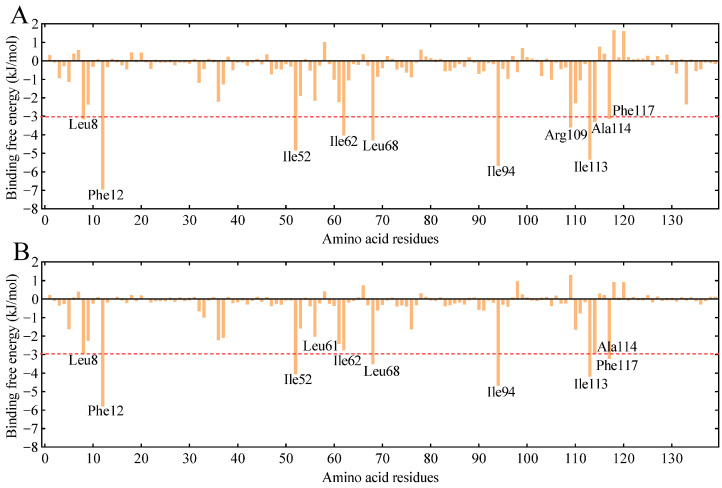
Total binding free energy contributions of each residue of GmolPBP2 to *Z*8-14:Ac (**A**) and *Z*8-12:Ac (**B**). The top ten residues with the lowest binding free energies are labeled. The red line signifies that the binding free energy value at this point is 3.0 kJ/mol.

GmolPBP2–*Z*8-14:Ac and GmolPBP2–*Z*8-12:Ac complexes were obtained by docking the *Z*8-14:Ac and *Z*8-12:Ac molecules into the binding pocket of GmolPBP2. Then, 200 nm MD simulations of the complexes were performed, followed by calculation of the theoretical binding free energy for each amino acid residue in the GmolPBP2–*Z*8-14:Ac and GmolPBP2–*Z*8-12:Ac complexes. A lower binding free energy indicated a stronger binding between the residue and the ligands. Ten residues exhibited binding free energies to *Z*8-14:Ac lower than −3.0 kJ/mol, including Phe12 (−6.960 kJmol), Ile94 (−5.674 kJ/mol), Ile113 (−5.350 kJ/mol), Ile52 (−4.841 kJ/mol), Leu68 (−4.305 kJ/mol), Ile62 (−4.033 kJ/mol), Arg109 (−3.596 kJ/mol), Ala114 (−3.299 kJ/mol), Leu8 (−3.150 kJ/mol), and Phe117 (−3.140 kJ/mol), suggesting their importance in *Z*8-14:Ac binding (Figure 5A). Notably, the NH2 atom in Arg109 formed a hydrogen bond (H-bond) with the carbonyl oxygen atom in the *Z*8-14:Ac acetyl group, with a bond distance of 2.9 Å (Figure 4D). Meanwhile, the phenyl side of Phe12 engaged in a π–alkyl interaction with the C14 atom of *Z*8-14:Ac, with a π–C14 distance of 4.5 Å (Figure 4D). Van der Waals forces were observed between the residues Leu68, Ile52, Ile94, and Ile113 and *Z*8-14:Ac (Figure 4F). The ten lowest binding free energies for GmolPBP2–*Z*8-12:Ac were found between Phe12 (−5.800 kJ/mol), Ile94 (−4.692 kJ/mol), Ile113 (−4.189 kJ/mol), Ile52 (−4.053 kJ/mol), Leu68 (−3.526 kJ/mol), 117Phe (−3.233), 114Ala (−2.968), 8Leu (−2.965), 62Ile (−2.758), 61Leu (−2.427) and *Z*8-12:Ac (Figure 5B). Like the GmolPBP2–*Z*8-14:Ac complex, van der Waals forces were the primary contributor to the binding of *Z*8-12:Ac (Figure 4G). However, differences arose from the hydrogen bonds: hydrogen bonds were formed between the side chain of Arg109 in GmolPBP2 and the acetyl of *Z*8-14:Ac, whereas no hydrogen bond was formed between GmolPBP2 and *Z*8-12:Ac (Figure 4E).

### 3.6. Validation of Key Amino Acid Residues for GmolPBP2 Binding to Z8-14:Ac

**Figure 6:**
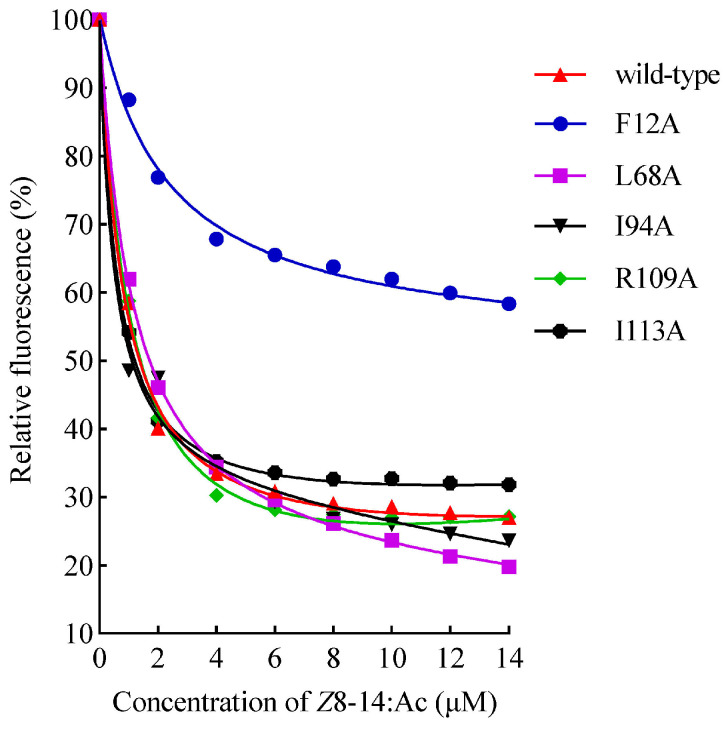
Curves of the binding of rGmolPBP2 (wild-type) and its five mutants to *Z*8-14:Ac. The fluorescence intensity after *Z*8-14:Ac displaced the 1–NPN probe bound to rGmolPBP2 is displayed as a percentage of the initial fluorescence intensity. The calculated *K_i_* values of rGmolPBP2 (wild-type) and mutants with *Z*8-14:Ac are listed in Table 5.

Based on the results of the aforementioned GmolPBP2–*Z*8-14:Ac docking analysis, five potential key residues (Phe12, Leu68, Ile94, Arg109, and Ile113) were individually substituted with Ala to generate the mutant proteins named F12A, L68A, I94A, R109A, and I113A, respectively. The results of SDS-PAGE showed that these mutants and wild-type rGmolPBP2 were all presented as inclusion bodies (Figure S3). The F12A mutant lost its affinity for binding *Z*8-14:Ac, as shown by the lack of *IC*_50_ and *K_i_* values for *Z*8-14:Ac when replacing the fluorescence probe, 1–NPN, in the F12A/1–NPN complex, suggesting that the π–alkyl interaction between Phe12 and the C14 atom of *Z*8-14:Ac was key to the binding affinity of GmolBPB2 for *Z*8-14:Ac (Figure 6). In contrast, the R109A mutant, which disrupted the hydrogen bond between Arg109 and the carbonyl oxygen of *Z*8-14:Ac, did not significantly affect the binding affinity. The L68A, I94A, and I113A mutants displayed binding affinities comparable to the wild-type GmolPBP2, suggesting that Leu68, Ile94, and Ile113 were not key amino acid residues that bind to *Z*8-14:Ac (Table 5).

## 4. Discussion

### 4.1. Z8-14:Ac Exhibits an Inhibitory Effect on OFM Males

Typically, species that share major sex pheromone components often use unique secondary components to generate species-specific signals [17,53,54]. Some secondary components serve as part of an intraspecific attractive message, while others may function as antagonists to closely related sympatric species [16,55,56]. *Z*8-14:Ac is a secondary sex pheromone component of female PFMs, and EAG tests indicated that *Z*8-14:Ac elicited EAG responses in both intraspecific PFM males and interspecific OFM males. Adding 1–30% of *Z*8-14:Ac to the PFM sex attractants (*Z*8-12:Ac/*E*8-14:Ac = 100:4, *m*/*m*) neither significantly increased nor inhibited the number of PFM males captured, but significantly reduced OFM males captured. Tòth et al. (1991) found that adding *Z*6-12:Ac (a sex pheromone analog) to OFM sex attractants caused a reduction in the capture of both OFM and PFM males, with a more pronounced effect on PFMs [57]. *Z*6-12:Ac acts as an attractive inhibitor for male PFMs, making OFM sex attractants more species-specific. *Heliotis peltigera*, *H*. *virescens*, and *H*. *assulta* are closely related sympatric species, and (*Z*)-9-tetradecenal (*Z*9-14:Al) is a sex pheromone component of female adult *H*. *peltigera* and *H*. *virescens*. Adding *Z*9-14:Al to the sex attractant of *H*. *assulta* notably decreased the capture of male *H*. *assulta* [57,58]. Guern et al. discovered that *Z*8-14:Ac and *Z*10-14:Ac were secondary sex pheromone components of female PFMs that did not increase *G*. *funebrana* catches but inhibited *G*. *molesta* [18]. Since that wonderful discovery, there have been few field trials specifically examining the individual roles of *Z*8-14:Ac and *Z*10-14:Ac in OFM inhibition, nor have the molecular mechanisms behind this inhibition been explored. At present, commercial PFM sex attractants contain only *Z*8-12:Ac and *E*8-12:Ac, indicating that *Z*8-14:Ac and *Z*10-14:Ac were not considered to have a synergistic effect during the development of these attractants. In the current study, field trials revealed that adding various amounts of *Z*8-14:Ac to a mix of sex attractants neither significantly increased nor inhibited the trapping of PFM males, but reduced the trap capture of OFM males by up to 96.54%. Additionally, as the concentration of *Z*10-14:Ac in the sex attractant increased, it exerted an inhibitory effect on both PFMs and OFMs. Therefore, it is hypothesized that the *Z*8-14:Ac of the PFM primarily functions to inhibit males of closely related sympatric species when added to a mixture of *Z*8-12:Ac and *E*8-12:Ac, thereby ensuring the species specificity of the sex pheromone.

### 4.2. GmolPBPs and GmolGOBPs Predominantly Expressed in OFM Adult Antennae

The expression patterns of insect OBPs are closely correlated with their physiological functions [59]. Three *GmolPBPs* and two *GmolGOBPs* were predominantly expressed in the antennae, suggesting their roles in chemoreception. There were variations in the expression levels of these five *GmolOBPs* in female and male adult antennae. *GmolPBP1*, *GmolPBP2*, and *GmolGOBP1* exhibited significantly higher expression levels in male antennae compared to female antennae. Conversely, *GmolPBP3* displayed significantly higher expression levels in female antennae than in male antennae. However, there was no significant difference in the expression level of *GmolGOBP2* between male and female antennae. Based on their expression patterns in the antennae, *GmolPBP1*, *GmolPBP2*, and *GmolGOBP1* are more likely to be involved in binding and transporting sex pheromones. Both PBPs and GOBPs in lepidopteron insects were highly expressed in the antennae of adults. However, the different expression levels in the antennae of both sexes depended on the species, such as in *Carposina sasakii* [60], *G*. *funebrana* [61], *Peridroma saucia* [62], and *Chilo suppressalis* [63,64]. What is more, the expression patterns of insect OBPs in different tissues seemed to be related to age after eclosion, mating status, and circadian rhythm [43,65,66]. In the present study, the expression levels of *GmolPBPs* and *GmolGOPBs* were only quantified in different tissues. Age-dependent, mating-dependent, and circadian rhythm-dependent expression patterns should be further illuminated.

### 4.3. GmolPBP2 Was the Most Likely Target for Binding Z8-14:Ac

*Z*8-14:Ac is a secondary PFM sex pheromone, as well as one of the sex pheromone components of *Ctenopseutis herana*, *C*. *obliquana*, *Planotortrix octo*, *P*. *excessana*, *Pandamis cerasana*, *Spilonota oceana*, and *S*. *laricana* [67,68,69]. In the current study, the ligand-binding assays revealed that rGmolPBP1, rGmolPBP2, rGmolPBP3, and rGmoGOBP2 all possess binding capabilities for *Z*8-14:Ac, indicating their overlapping functions in binding this component. Among these four rGmolPBPs/rGmolGOBPs, rGmolPBP2 exhibited the strongest binding affinity for *Z*8-14:Ac, potentially functioning as the primary GmolOBP responsible for perceiving and transporting Z8-14:Ac. Four PBPs (GfunPBP1.1, GfunPBP1.2, GfunPBP2, and GfunPBP3) and three GOBPs (GfunGOBP1, GfunGOBP2, and GfunGOBP3) were identified in PFMs antennae. rGfunPBP1.1, rGfunPBP1.2, and rGfunGOBP3 exhibited significantly stronger binding affinities for *Z*8-14:Ac than rGfunPBP2, rGfunPBP3, rGfunGOBP1, and rGfunGOBP2, indicating that the PBPs/GOBPs of PFMs and OFMs differ in their binding to *Z*8-14:Ac [2,61]. The sex pheromones released by OFM females consist of *Z*8-12:Ac, *E*8-12:Ac, *Z*8-12:OH, and 12:OH, with GmolPBP2 showing a binding preference for *Z*8-12:Ac and *E*8-12:Ac [43], GmolGOBP2 exhibiting the strongest binding affinity for 12:OH [44], and GmolPBP1 demonstrating a preference for binding to *Z*8-12:OH [45]. The strong binding affinity of GmolPBP2 for *Z*8-14:Ac may be related to *Z*8-14:Ac sharing a similar chemical structure and functional group with *Z*8-12:Ac. However, direct evidence of GmolPBP2 binding to *Z*8-14:Ac needs further analysis using protein crystallography to fully elucidate the interactions of the GmolPBP2–Z8-14:Ac complex. In addition to chemoreception, insect PBPs/GOBPs are involved in binding and transporting “non-semiochemical” ligands, such as BmorPBP1 and BmorGOBP2 in *Bombyx mori*, which bind vitamins, insecticides, and juvenile hormones [65,70]. *Athetis lepigone* AlepPBP2, AlepPBP3, and AlepGOBP2 have high binding affinities for insecticide phoxim and play an important role in phoxim adaptation [71]. *Glyphodes pyloalis* GpylPBP1 exhibits a strong binding affinity for chlorpyrifos and phoxim, with the Phe12, Ile52, and Phe118 residues being crucial binding sites for both insecticides [40]. Further research is needed to explore the functions of GmolPBPs/GmolGOBPs beyond chemoreception.

### 4.4. Key Amino Acid Residues and Interaction Forces of GmolPBP2 Binding to Z8-14:Ac

Classical OBPs possess a binding pocket formed by six typical α-helices. OBPs possess the capability to bind sex pheromones and other volatile semiochemicals as the molecules of these compounds enter the binding pocket and form specific binding interactions with the residues within the binding cavities [72,73,74]. The interactions between OBPs and odorant ligands encompass hydrogen bonds, electrostatic forces, van der Waals, π-π interactions, and π–alkyl interactions [75,76,77]. Hydrogen bonds frequently serve as the primary interaction force between insect PBPs and sex pheromone molecules, as observed in various examples such as *Bombyx mori* BmorPBP1 and bombykol [72], *Epiphyas Postvittana* EposPBP3 and *E*11-14:OH [78], and *Apis mellifera* ASP1 and 9-keto-2(E)-decenoic acid (9-ODA) [79]. Tian et al. analyzed the binding interactions of the GmolPBP2–*Z*8-12:Ac complex [80]. In our study, the GmolPBP2 sequence differs by one additional amino acid on the N-terminus due to the use of a different version of SignalP software (version 5.0). Additionally, Tian et al. used AMMBER (v.12) and we used GROMACS (v. 2023.3) for molecular dynamics simulations of GmolPBP2–Z8-12:Ac [80]. As a result, the calculated binding free energies in this study of GmolPBP2–*Z*8-12:Ac and hydrogen bonds were based on the GROMACS results. The five lowest energies of residues for GmolPBP2–*Z*8-12:Ac were found at Phe12 (−5.800 kJ/mol), Ile94 (−4.692 kJ/mol), Ile113 (−4.189 kJ/mol), Ile52 (−4.053 kJ/mol), and Leu68 (−3.526 kJ/mol). Likewise, the five lowest energies for GmolPBP2–*Z*8-14:Ac were observed at Phe12 (−7.352 kJ/mol), Ile94 (−5.863 kJ/mol), Ile113 (−4.103 kJ/mol), Ala114 (−4.071 kJ/mol), and Leu68 (−3.897 kJ/mol). Van der Waals forces were the primary contributors to the binding between GmolPBP2 and both sex pheromone components. However, differences arose in the hydrogen bonds: no hydrogen bond was found in GmolPBP2–*Z*8-12:Ac, while a hydrogen bond was formed between the side chain of Arg109 in GmolPBP2 and the carbonyl oxygen of *Z*8-14:Ac. According to the site-mutagenesis results, only the F12A mutant lost affinity for *Z*8-14:Ac, suggesting that Phe12 was the key amino acid residue for GmolPBP2 binding to *Z*8-14:Ac. The π–alkyl interaction, formed between the phenyl sidechain of Phe12 and the C14 atom of *Z*8-14:Ac, was identified as the primary interaction force in GmolPBP2’s binding to *Z*8-14:Ac, whereas the H-bond between the NH2 atom of Arg109 and the carbonyl oxygen atom in *Z*8-14:Ac was not. Li et al. discovered that GfunPBP1.1 of the PFM preferentially bound to *Z*8-14:Ac, whereas a hydrogen bond was observed between the OA atom of Ser56 and the O atom from the acetoxy group of *Z*8-14:Ac [2]. Moreover, a π–alkyl interaction formed between the phenyl of Trp37 and the C14 atom of *Z*8-14:Ac. These results imply that, even among closely related species, there are variations in the residues and binding forces of PBPs when binding to the same ligand.

## Figures and Tables

**Table 1:** Captures of PFM and OFM males in a peach orchard using delta traps with varying amounts of *Z*8-14:Ac added to a mixture of *Z*8-12:Ac and *E*8-12:Ac.

Pheromone Loadings (μg)			Mass Ratio of *Z*8-12:Ac, *E*8-12:Ac, and *Z*8-14:Ac	Total PFM Males/Trap	Total OFM Males/Trap	Inhibition Rate of OFM Males (%)
*Z*8-12:Ac	*E*8-12:Ac	*Z*8-14:Ac				
500	20	0	100:4:0	302.20 ± 51.88 a	111.87±6.24 a	0.00
500	20	5	100:4:1	387.73 ± 18.31 a	46.08±3.25 b	58.81
500	20	10	100:4:2	287.22 ± 7.96 a	40.89±2.35 b	63.45
500	20	25	100:4:5	314.53 ± 26.14 a	14.82±1.47 c	86.75
500	20	50	100:4:10	331.58 ± 17.46 a	9.04±1.08 c	91.92
500	20	75	100:4:15	297.81 ± 4.75 a	9.55±1.09 c	91.46
500	20	100	100:4:20	285.62 ± 6.55 a	9.67±1.52 c	91.36
500	20	125	100:4:25	352.89 ± 32.42 a	6.86±0.24 c	93.87
500	20	150	100:4:30	348.30 ± 22.77 a	7.96±1.19 c	92.88

Note: Data in the table are presented as mean ± *SEM* (*n* = 3). The mass ratios of *E*8-12:Ac and *Z*8-14:Ac were calculated based on a *Z*8-12:Ac content of 100%. Different lowercase letters in each column represent significant differences using one-way ANOVA followed by Tukey’s HSD test (*α* = 0.05).

**Table 2:** Captures of PFM and OFM males in a pear orchard using delta traps with varying amounts of *Z*8-14:Ac added to a mixture of *Z*8-12:Ac and *E*8-12:Ac.

Pheromone Loadings (μg)			Mass Ratio of *Z*8-12:Ac, *E*8-12:Ac, and *Z*8-14:Ac	Total PFM Males/Trap	Total OFM Males/Trap	Inhibition Rate of OFM Males (%)
*Z*8-12:Ac	*E*8-12:Ac	*Z*8-14:Ac				
500	20	0	100:4:0	56.49 ± 3.80 a	169.47 ± 9.83 a	0.00
500	20	5	100:4:1	64.07 ± 6.89 a	59.24 ± 10.14 b	65.04
500	20	10	100:4:2	45.47 ± 6.64 a	61.31 ± 7.21 b	63.82
500	20	25	100:4:5	43.06 ± 0.34 a	21.36 ± 6.22 c	87.40
500	20	50	100:4:10	53.73 ± 7.04 a	12.05 ± 0.91 c	92.89
500	20	75	100:4:15	48.57 ± 6.31 a	8.95 ± 2.69 c	94.72
500	20	100	100:4:20	42.36 ± 8.46 a	12.74 ± 6.57 c	92.48
500	20	125	100:4:25	57.52 ± 21.06 a	5.86 ± 1.50 c	96.54
500	20	150	100:4:30	72.67 ± 12.44 a	10.33 ± 2.98 c	93.90

Note: Data in the table are presented as mean ± *SEM* (*n* = 3). The mass ratios of *E*8-12:Ac and *Z*8-14:Ac were calculated based on a *Z*8-12:Ac content of 100%. Different lowercase letters in each column represent significant differences using one-way ANOVA followed by Tukey’s HSD test (*α* = 0.05).

**Table 3:** Captures of PFM and OFM males in a pear orchard using delta traps with varying amounts of *Z*10-14:Ac added to a mixture of *Z*8-12:Ac and *E*8-12:Ac.

Pheromone Loadings (μg)			Mass Ratio of *Z*8-12:Ac, *E*8-12:Ac, and *Z*10-14:Ac	Total PFM Males/Trap	Total OFM Males/Trap	Inhibition Rate of OFM Males (%)
*Z*8-12:Ac	*E*8-12:Ac	*Z*10-14:Ac				
500	20	0	100:4:0	142.55 ± 11.16 a	193.28 ± 60.19 a	0.00
500	20	25	100:4:5	121.32 ± 12.10 ab	125.55 ± 14.47 ab	35.04
500	20	50	100:4:10	101.39 ± 7.72 b	97.92 ± 10.31 bc	49.34
500	20	100	100:4:20	102.73 ± 10.21 b	83.33 ± 9.82 cd	56.89
500	20	150	100:4:30	66.08 ± 8.27 c	33.56 ± 2.24 e	82.64
500	20	250	100:4:50	62.46 ± 5.63 c	31.28 ± 3.22 e	83.82
500	20	350	100:4:70	51.35 ± 7.21cd	37.49 ± 3.45 e	80.70
500	20	450	100:4:90	41.24 ± 5.10 d	23.64 ± 1.23 e	87.77
500	20	550	100:4:110	42.33 ± 3.95 d	29.69 ± 2.18 e	84.64

Note: Data in the table are presented as mean ± *SEM* (*n* = 3). The mass ratios of *E*8-12:Ac and *Z*10-14:Ac were calculated based on a *Z*8-12:Ac content of 100%. Different lowercase letters in each column represent significant differences using one-way ANOVA followed by Tukey’s HSD test (*α* = 0.05).

**Table 4:** Binding affinities of rGmolPBPs and rGmolGOBPs for *Z*8-14:Ac.

Protein Name	*K_d_* (μM)	*IC*_50_ (μM)	*K_i_* (μM)
GmolPBP1	1.29 ± 0.02	2.93 ± 0.07	1.66 ± 0.04 c
GmolPBP2	1.09 ± 0.02	1.26 ± 0.04	0.66 ± 0.02 d
GmolPBP3	11.24 ± 0.08	2.78 ± 0.32	2.56 ± 0.27 b
GmolGOBP1	5.76 ± 0.01	>14	>14 a
GmolGOBP2	4.32 ± 0.02	3.53 ± 0.22	2.86 ± 0.18 b

Note: *IC*_50_: the concentration of *Z*8-14:Ac when replacing 1−NPN to reduce the initial fluorescence intensity of the rGmolPBP(rGmolGOBP)/1−NPN complex to 50%; *K_d_*: the dissociation constants of each rGmolPBP (rGmolGOBP) binding to 1−NPN; *K_i_*: the inhibition constants of *Z*8-14:Ac competitive binding with each GmolPBP (GmolGOBP) from the protein/1−NPN complex. Data in the table are presented as mean ± *SE* (*n* = 3). Different lowercase letters after the data in the same column indicate significant differences in the affinities of different recombinant proteins for binding to *Z*8-14:Ac (*p* < 0.05, one-way ANOVA followed by Tukey’s HSD test).

**Table 5:** Binding affinities of wild-type and mutants of GmolPBP2 to *Z*8-14:Ac.

Protein Name	*IC*_50_ (μM)	*K_i_* (μM)
wild-type	1.260 ± 0.036	0.660 ± 0.019
F12A	>14	>14
L68A	1.129 ± 0.026	0.589 ± 0.007
I94A	1.082 ± 0.008	0.564 ± 0.004
R109A	1.257 ± 0.029	0.655 ± 0.015
I113A	0.980 ± 0.038	0.520 ± 0.020

Note: *IC*_50_: the concentration of *Z*8-14:Ac when replacing 1–NPN to reduce the initial fluorescence intensity of rGmolPBP2 (and its mutants)/1–NPN complex to 50%; *K_i_*: the inhibition constants of *Z*8-14:Ac competitive binding with wild-type GmolPBP2 and each mutant from the protein/1–NPN complex. Data in the table are presented as mean ± *SE* (*n* = 3).

## Data Availability

Data are available upon request from Guangwei Li.

## Supplementary Materials

The following supporting information can be downloaded at https://www.mdpi.com/article/10.3390/insects15120918/s1, Figure S1: SDS-PAGE results showing the expressed products and purified rGmolPBP1 (A), rGmolPBP2 (B), rGmolPBP3 (C), rGmolGOBP1 (D), and rGmolGOBP2 (E) of OFMs. Figure S2: The Ramachandran plots of simulated GmolPBP2 display the residues in the most favored regions, additional allowed regions, and disallowed regions. Figure S3: SDS-PAGE of the rGmolPBP2 (wild-type) (A) and the mutants F12A (B), L68A (C), I94A (D), R109A (E), and I113A (F). Table S1: Specific primers used for gene expression level detection, prokaryotic expression, and site-directed mutagenesis. Table S2: The 33 amino acids that constitute the binding pocket of GmolPBP2.
