# Supplementary material for: Functional Role of Odorant-Binding Proteins in Response to Sex Pheromone Component Z8-14:Ac in Grapholita molesta (Busck)
Source: Insects. 2024 Nov 25;15(12):918. doi: 10.3390/insects15120918 (PMC11678869; doi:10.3390/insects15120918)
Supplement: Supplementary file 1 [file insects-15-00918-s001.zip › Table S1.pdf]

Table S1. Specific primers used for gene expression levels detection, prokaryotic expression, and site-directed mutagenesis

Primers	Primer sequences (5′-3′)	Product sizes (bp)
qGmolPBP1-F	CTTTGCCATGAAACATGGTG	For detected tissue expression of five *GmolOBP* genes
qGmolPBP1-R	GCATGCACTGGTCTTCTTGA	
qGmolPBP2-F	AGAAACAGAACACTGGCGCT	
qGmolPBP2-R	AACACCGTCTCCATACTCGG	
qGmolPBP3-F	GACACTGGTTGTGCCATTATATGC	
qGmolPBP3-R	CATGAGTCTTCTCGCAGGCGTGGA	
qGmolGOBP1-F	CAAGTTCATCAAGAGTTTCCCTAA	
qGmolGOBP1-R	CAAGCATCAGCCTCCATAATAA	
qGmolGOBP2-F	GTTGGAGCAGTGTCGGGAGGA	
qGmolGOBP2-R	AGCGGATAGGACATCACCTTTGG	
Gmolβ-actin-F	CTTTCACCACCACCGCTG	
Gmolβ-actin-R	CGCAAGATTCCATACCCA	
GmolEF1-α-F	AGGAGATCGAGCAACAGGAA	
GmolEF1-α-R	CACGACTCTCGGGACTTCTC	
xGmolPBP1-F	CCGGAATTCTCGCAGCAGGTGATAAAAG	Expression of protein
xGmolPBP1-R	CCCAAGCTTTTAAACTTCAGCTAGAAC	
xGmolPBP2-F	CGGGATCCTCGGCAGACATTATGAA	
xGmolPBP2-R	CCCAAGCTTCTACGACGGCTTGACTT	
xGmolPBP3-F	CGGGATCCATAGAATTGTCATCGGA	
xGmolPBP3-R	CCCTCGAGGGTTACATCTCAGTGAGAA	
xGmolGOBP1-F	CGGGATCCACCCAGGAGGTGCTGAA	
xGmolGOBP1-R	CCCAAGCTTGGGTCAAGCATCAGCCTCCA	
xGmolGOBP2-F	CGGGATCCGGCCGGATGGTAGATGGTAC	
xGmolGOBP2-R	CCCAAGCTTGGGTCAGTATTTCTCCAGTACAG	
F12A-forward	CTACAGGAGCTGCGACGGCGCTAGATAAGTG	For site-specific mutagenesis
F12A-reverse	GTCGCAGCTCCTGTAGTTAATTTCTTCATAATG	
L68A-forward	CGCCAAAGCACATCACAAGAACGCTCATGAGTTC	
L68A-reverse	GTGATGTGCTTTGGCGTCATCGTCAATGAGGTCG	
I94A-forward	GTAGCCATGGCTCACGAGTGCGAGAAACAGAACAC	
I94A-reverse	CTCGTGAGCCATGGCTACCAGTTGCTTGGCTAGGTC	
R109A-forward	CTGCATCGCGACCCTGGGCATCGCCAAGTGCTTCAG	
R109A-reverse	CCAGGGTCGCGATGCAGTCATCAGCGCCAGTGTTC	
I113A-forward	CTGGGCGCCGCCAAGTGCTTCAGAACGAAGATCC	
I113A-reverse	CACTTGGCGGCGCCCAGGGTCCTGATGCAGTCATC	
