# Supplementary material for: Functional Role of Odorant-Binding Proteins in Response to Sex Pheromone Component Z8-14:Ac in Grapholita molesta (Busck)
Source: Insects. 2024 Nov 25;15(12):918. doi: 10.3390/insects15120918 (PMC11678869; doi:10.3390/insects15120918)
Supplement: Supplementary file 1 [file insects-15-00918-s001.zip › Table S2.pdf]

Table S2. The 33 amino acids that constitute the binding pocket of GmolPBP2.

Name of amino acid residues	Hydrophilic/Hydrophobic
Met5	Hydrophobic
Leu8	Hydrophobic
Thr9	Hydrophilic
Phe12	Hydrophobic
Phe33	Hydrophobic
Phe36	Hydrophobic
Trp37	Hydrophobic
Ile52	Hydrophobic
Leu53	Hydrophobic
Met55	Hydrophobic
Ala56	Hydrophobic
Leu61	Hydrophobic
Ile62	Hydrophobic
Ala66	Hydrophobic
Lys67	Hydrophilic
Leu68	Hydrophobic
Ala73	Hydrophobic
His74	Hydrophilic
Phe76	Hydrophobic
Ala77	Hydrophobic
Leu86	Hydrophobic
Ala87	Hydrophobic
Leu90	Hydrophobic
Ala91	Hydrophobic
Ile94	Hydrophobic
Glu98	Hydrophilic
Arg109	Hydrophilic
Thr110	Hydrophilic
Ile113	Hydrophobic
Ala114	Hydrophobic
Phe117	Hydrophobic
Arg118	Hydrophilic
Val133	Hydrophobic
