# Supplementary figures and images for: Functional Role of Odorant-Binding Proteins in Response to Sex Pheromone Component Z8-14:Ac in Grapholita molesta (Busck)
Source: Insects. 2024 Nov 25;15(12):918. doi: 10.3390/insects15120918 (PMC11678869; doi:10.3390/insects15120918)

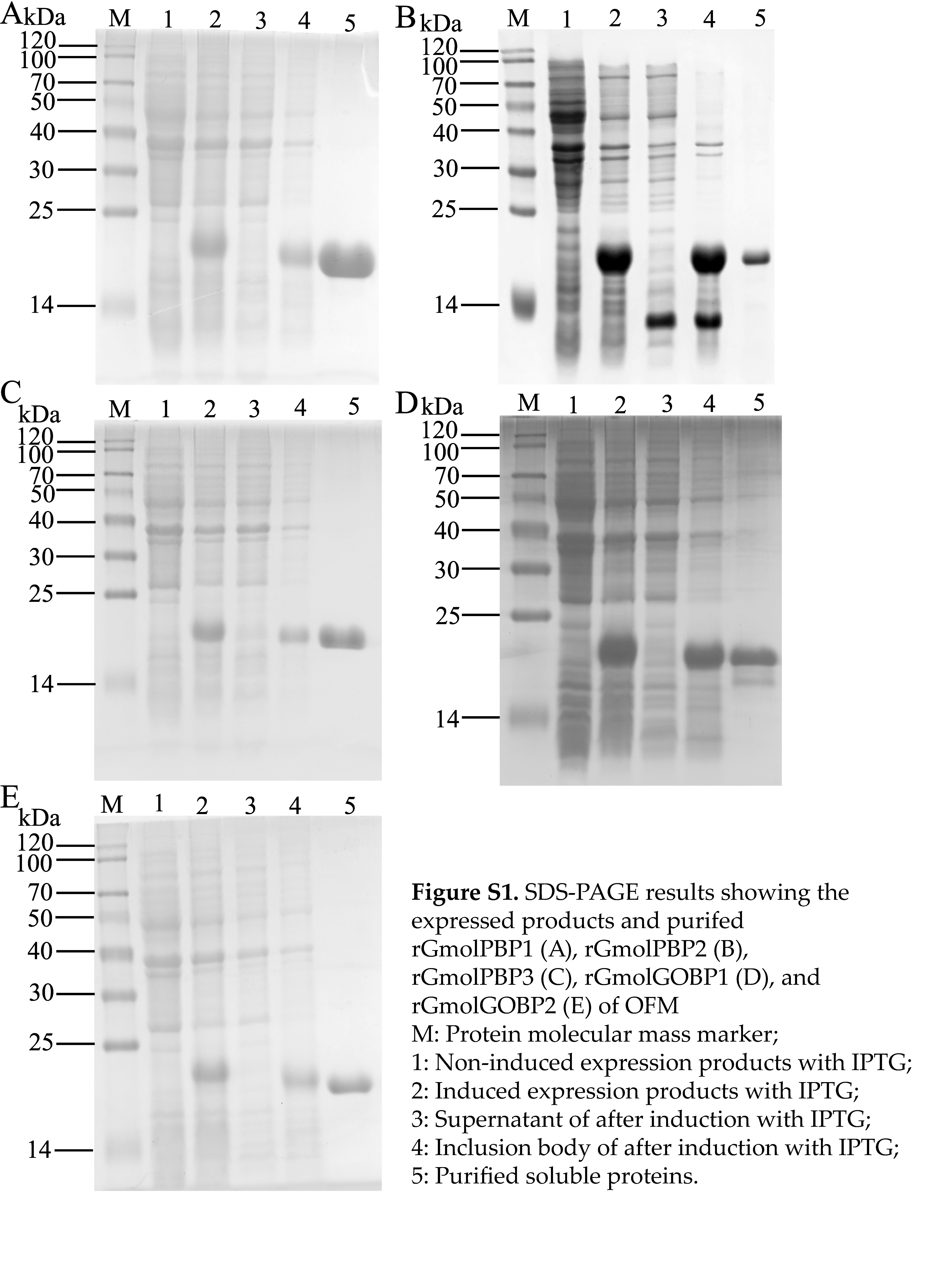

Supplement: Supplementary file 1 [file insects-15-00918-s001.zip › Figure S1.jpg]

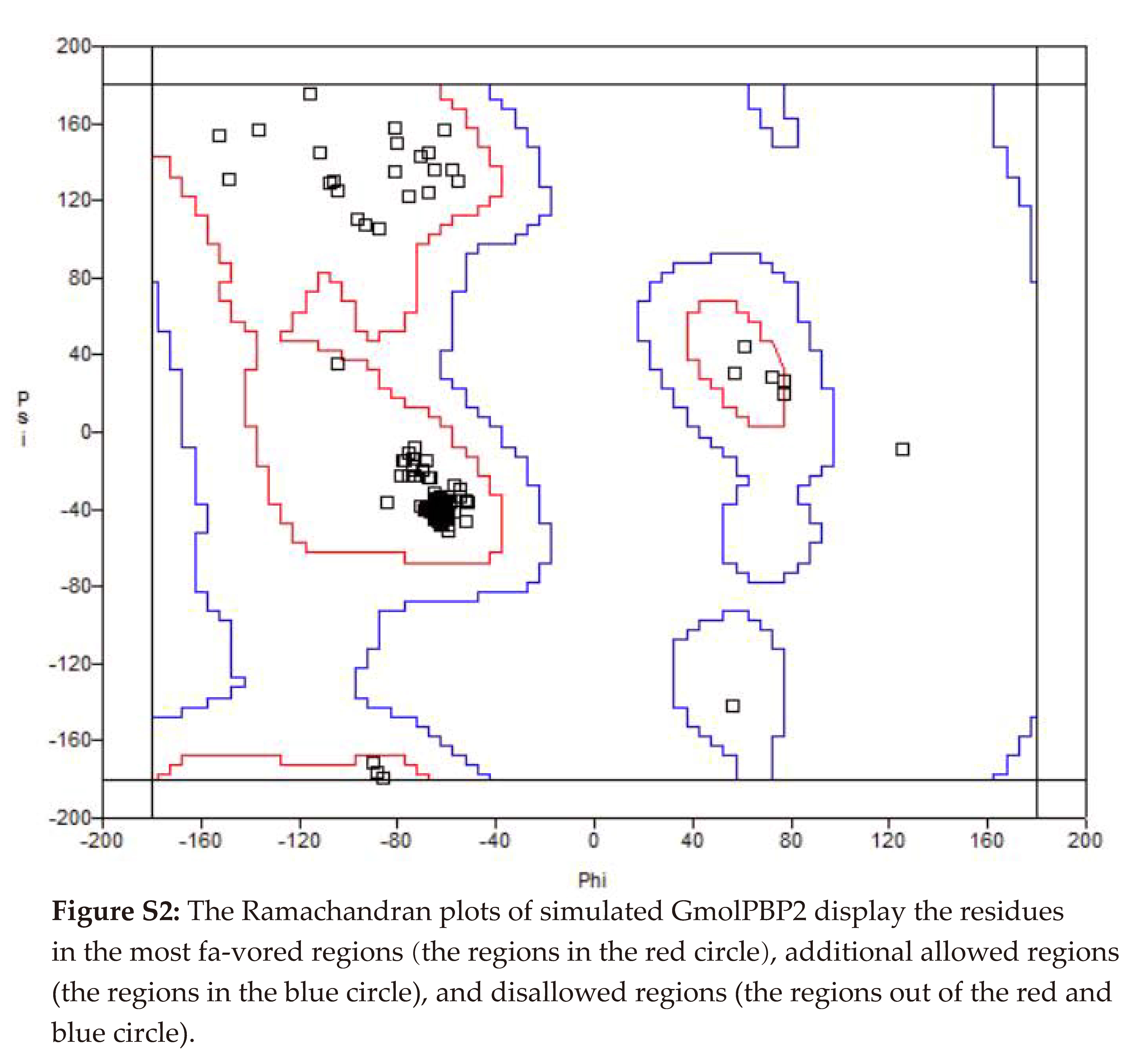

Supplement: Supplementary file 1 [file insects-15-00918-s001.zip › Figure S2.jpg]

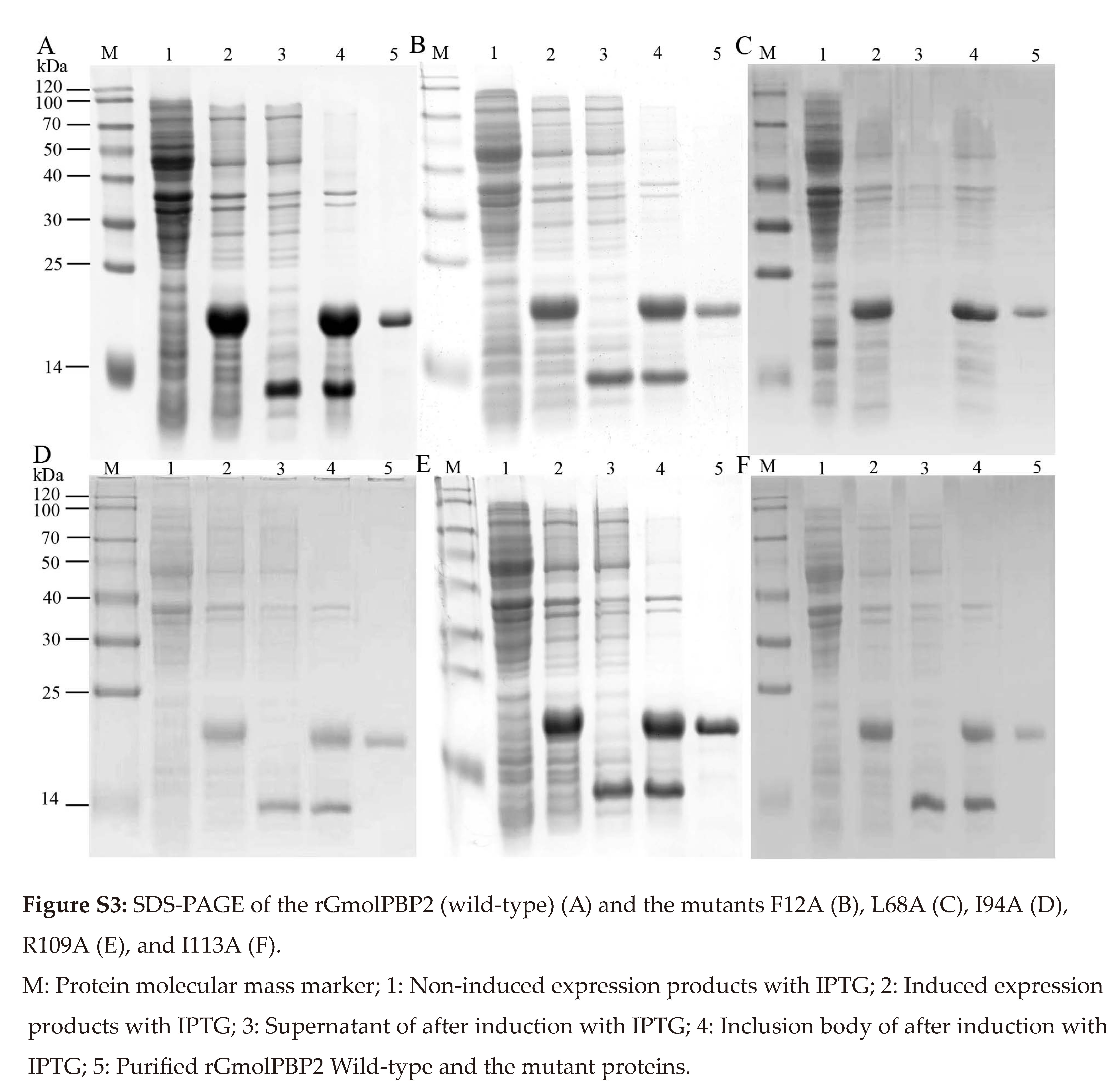

Supplement: Supplementary file 1 [file insects-15-00918-s001.zip › Figure S3.jpg]
